# Supplementary material for: Identification of an Immune-Related Gene Signature for Prognostic Prediction in Glioblastoma: Insights from Integrated Bulk and Single-Cell RNA Sequencing
Source: Cancers (Basel). 2025 May 28;17(11):1799. doi: 10.3390/cancers17111799 (PMC12153616; doi:10.3390/cancers17111799)
Supplement: Supplementary file 1 [file cancers-17-01799-s001.zip › cancers-3656513-supplementary.pdf]

Supplementary Figures

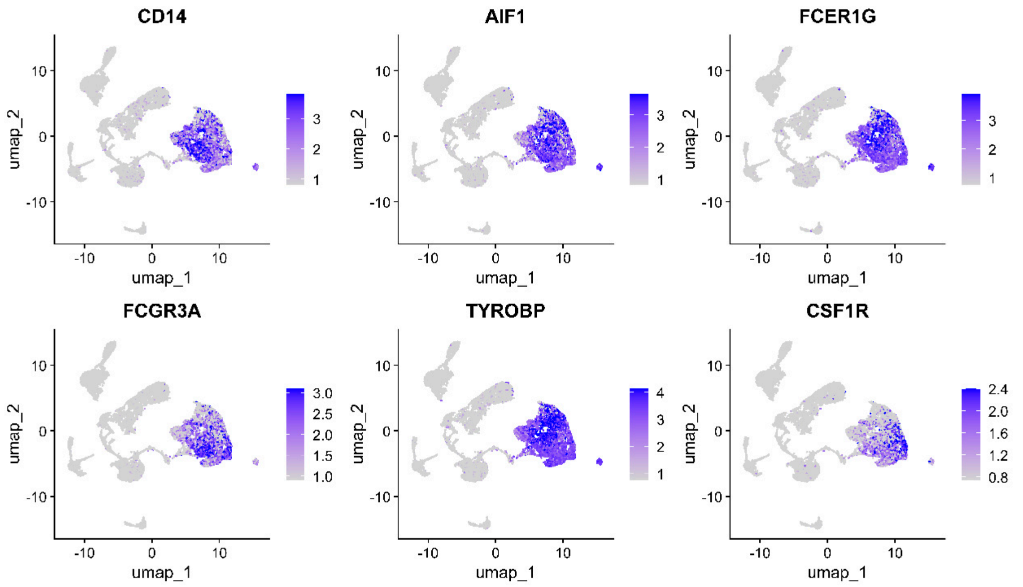

Supplementary Figure S1. Macrophage clusters were identified based on the expression of canonical marker genes, including *CD14, AIF1, FECR1G, FCGR3A, TYROBP,* and *CSF1R*.

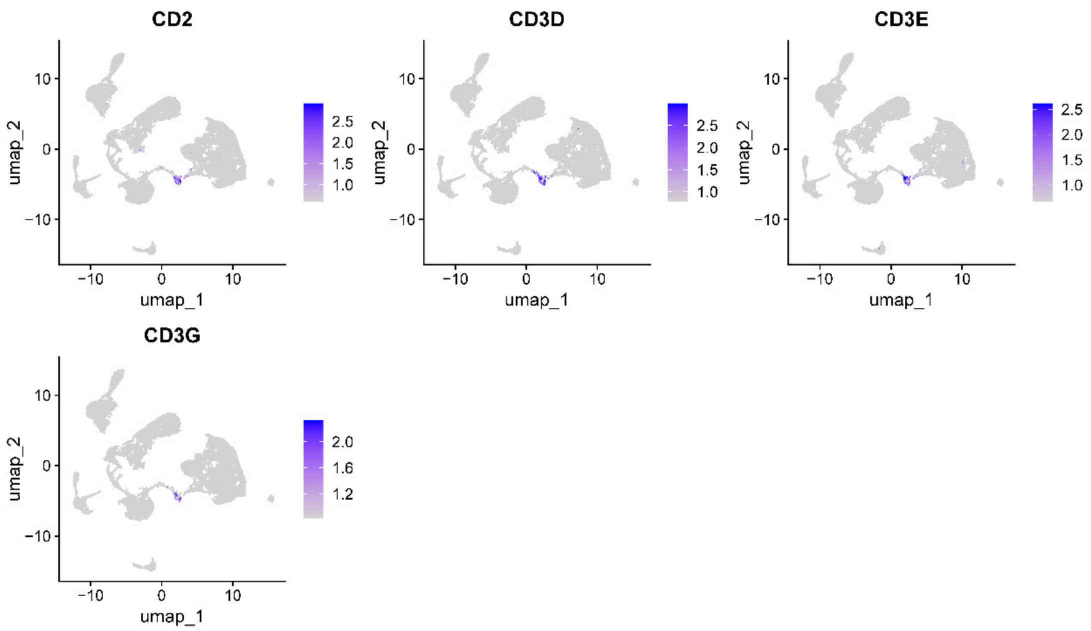

Supplementary Figure S2. T cell clusters were identified according to the expression of canonical marker genes, including *CD2, CD3D, CD3E* and *CD3G*.

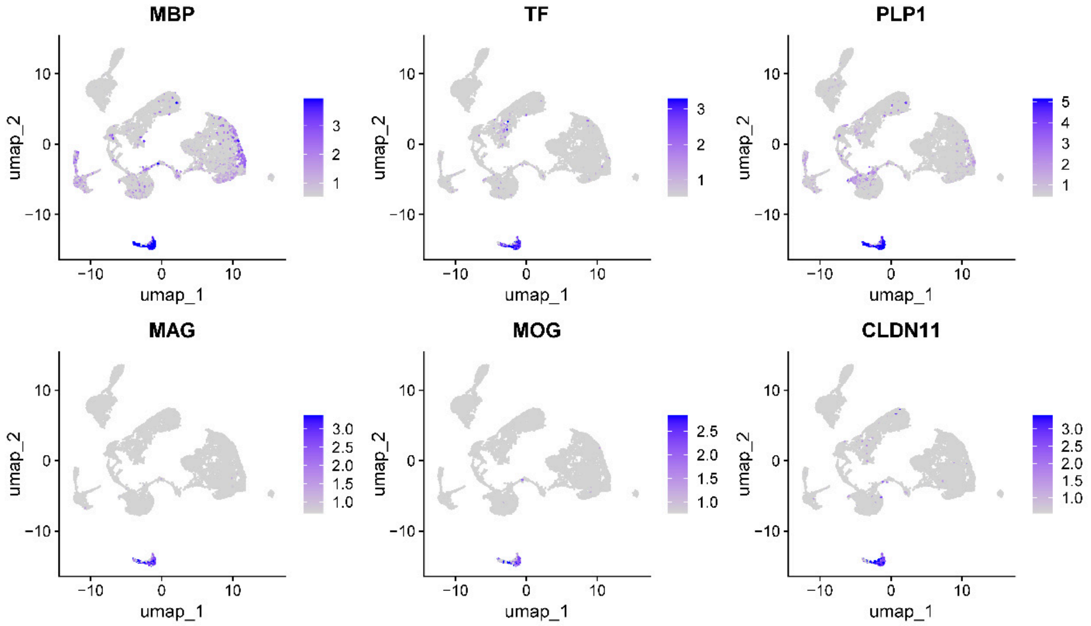

Supplementary Figure S3. Oligodendrocyte clusters were annotated based on the expression of classical marker genes such as *MBP, PLP1, TF, MAG, MOG,* and *CLDN11*.

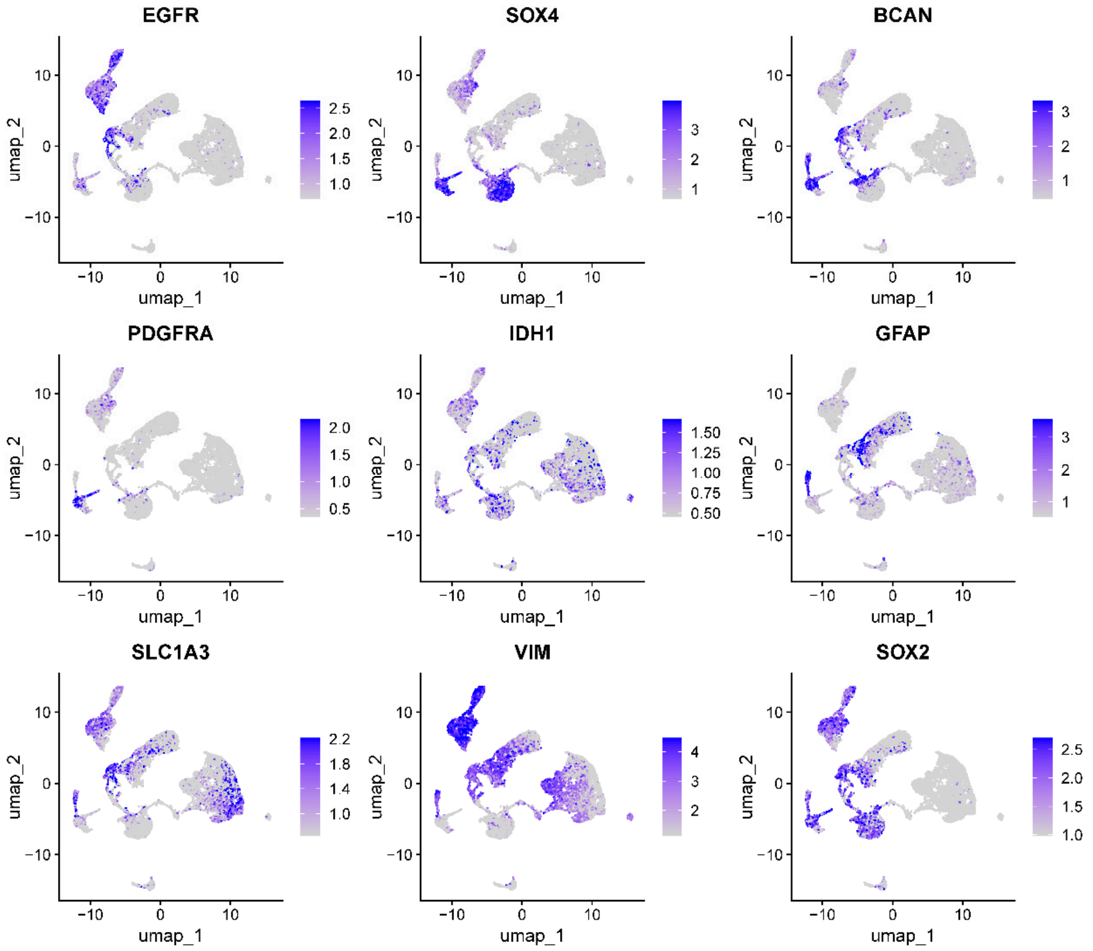

Supplementary Figure S4. Tumor clusters were determined by the expression of established marker genes, including *EGFR, SOX4, BCAN, PDGFRA, IDH1, GFAP, SLC1A3, VIM,* and *SOX2*.

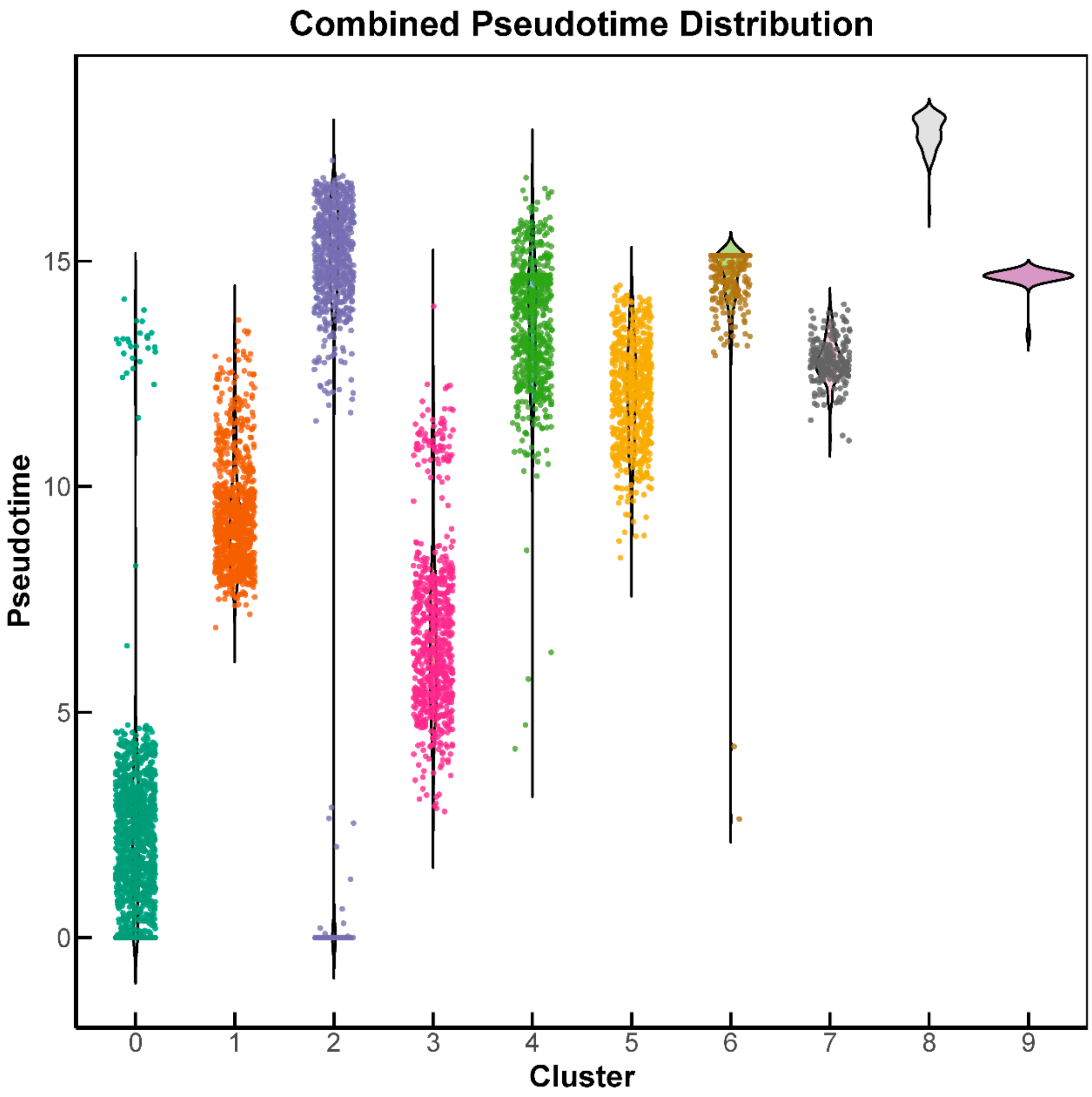

Supplementary Figure S5. The starting cluster was determined based on the distribution of cells along the inferred pseudotime trajectory.

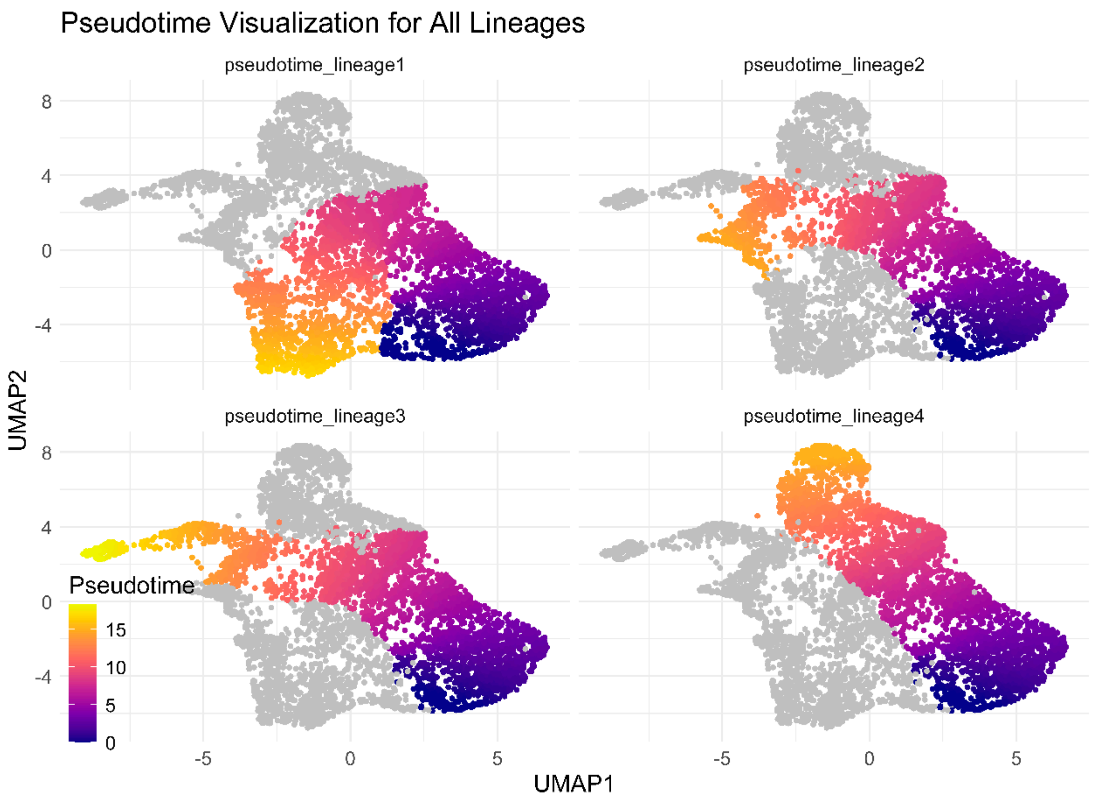

Supplementary Figure S6. Trajectory analysis revealed two additional transition-state macrophage subtypes, designated as Pre-TAM and Trans-TAM, representing intermediate stages during macrophage differentiation.
